# Disentangling Cognitive Load From Visual Reflexes: An Iso-Luminant Framework for Virtual Reality (VR)-Based Pupillometry

**DOI:** 10.7759/cureus.101950

**Published:** 2026-01-20

**Authors:** Umut Yilmaz, Cemre Karadeniz, Mert Talha Yener, Musa Atas, Eser Sagaltici

**Affiliations:** 1 Centre for Autonomous and Intelligent Systems, University of Huddersfield, Huddersfield, GBR; 2 Neuroscience, University of Padua, Padua, ITA; 3 Research and Development, VoctoR Health, Istanbul, TUR; 4 Computer Engineering, Siirt University, Siirt, TUR; 5 Psychiatry, Antalya City Hospital, University of Health Sciences, Antalya, TUR

**Keywords:** cognitive load, eye tracking, iso-luminant paradigm, locus coeruleus, pupillary light reflex, pupillometry, task-evoked pupillary response, virtual reality

## Abstract

Pupillometry is a robust, non-invasive indicator of cognitive load; however, its application in virtual reality (VR) is compromised by luminance-driven pupillary light reflexes (PLR) that mask subtle task-evoked pupillary responses (TEPR). We introduce a strict iso-luminant VR protocol to isolate cognitive load-related autonomic responses. The framework comprises a PLR validation phase establishing reflex dynamics via controlled luminance alternations, followed by a cognitive isolation phase where visual luminance is clamped constant and cognitive load is modulated exclusively through auditory tasks. Analysis of 22 sessions confirmed PLR sensitivity (mean amplitude: 1.52 ± 0.33 mm, constriction velocity: 19.1 ± 11.2 mm/s). Critically, under iso-luminant conditions, TEPR was successfully isolated with significant dilation during high-load versus low-load conditions (mean difference: 0.38 ± 0.28 mm). The framework differentiated temporal signatures: PLR exhibited rapid velocity (19.1 mm/s), while cognitive load induced gradual dilation (3.86 mm/s), demonstrating a response velocity increased by a factor of 5.2 compared with the cognitive condition. This temporal dissociation confirms that pupillary changes under iso-luminant conditions reflect cognitive processing through the locus coeruleus-norepinephrine (LC-NE) pathway rather than brainstem-mediated light reflexes. The methodology establishes a reproducible foundation for VR-based neuro-ergonomic research with potential applications in attention-deficit/hyperactivity disorder (ADHD) assessment and neurodegenerative disease monitoring.

## Introduction

Pupil diameter serves as a window into the autonomic nervous system, reflecting both reflexive responses to environmental stimuli and cognitive-emotional states [1]. The pupil's dual innervation - parasympathetic constriction via the Edinger-Westphal nucleus and sympathetic dilation mediated by the locus coeruleus-norepinephrine (LC-NE) system - provides access to distinct neural pathways through a single non-invasive measure [2,3].

The pupillary light reflex (PLR) represents a brainstem-mediated response wherein pupil diameter modulates rapidly in response to luminance changes, typically completing within 200-500 milliseconds [4]. In contrast, task-evoked pupillary responses (TEPR) associated with cognitive load operate through the LC-NE pathway, producing more gradual dilations that manifest over 1-3 seconds [5,6]. This temporal distinction - rapid PLR versus gradual TEPR - is neuroanatomically grounded and represents a key validation criterion for isolating cognitive effects.

The neuroanatomical dissociation is fundamental: the PLR is a rapid reflex loop involving the pretectal olivary nucleus and the Edinger-Westphal nucleus, whereas TEPR is a slower, poly-synaptic modulation driven by the LC-NE system's projections to the dilator muscle. This study operationalizes this difference as a 'velocity ratio' to provide a quantitative benchmark for isolation success.

Virtual reality (VR) systems with integrated eye-tracking offer powerful platforms for human factors research [7,8]. However, dynamic VR environments produce continuous luminance variations that trigger PLR, contaminating cognitive load measurements. Previous approaches employing post-hoc luminance correction assume linear PLR responses and stable temporal dynamics, assumptions frequently violated in complex environments [9,10]. The present work introduces an alternative: a strict iso-luminant framework that eliminates luminance confounds at the source, enabling direct TEPR measurement. It is important to note that this scope is intended primarily as a technical and methodological validation rather than a broad cognitive or clinical investigation.

## Technical report

The analytical backend was engineered using a modular Python 3 pipeline, leveraging Pandas 2.0+ for vectorized time-series manipulation and SciPy for precision signal processing. For rapid, iterative validation, the framework integrates Streamlit and Plotly, establishing a scalable, reproducible neuro-informatics workflow.

Experimental design

The iso-luminant paradigm was developed at VoctoR Health and consists of a single 7-8 minute session implemented in Unity-based VR using an HTC Focus Vision headset with integrated eye-tracking (approximately 90 Hz sampling rate). The system was developed in Unity 6000.0.58f2 Long-Term Support (LTS). To ensure precise luminance control independent of viewing angle, the VR environment utilized 'Unlit' shaders, bypassing the standard lighting pipeline to prevent specular highlights or shadows. Thus, visual stimuli were modulated strictly by programmed RGB changes. Note that RGB values were utilized as proxies for relative luminance levels; absolute photometric calibration was not performed. Data acquisition utilized the Vive OpenXR SDK, capturing binocular pupil diameter, eye openness, and gaze position. To ensure environmental control, all sessions were conducted in a dimly lit room (controlled < 10 lux) to prevent external light leakage through the headset gasket from influencing the baseline pupillary state.

Participants and duration

A total of 22 healthy volunteers were recruited for the study based on normal or corrected-to-normal vision and no history of neurological disorders. Mean age was 26.1 years (SD = 4.2), with 12 females and 10 males. The mean duration of the experimental sessions was approximately 7.5 minutes.

Pre-experimental procedure

Prior to data collection, a standard 5-point eye-tracking calibration was performed via the headset's integrated system interface to ensure gaze accuracy. Following successful calibration, the experimental protocol proceeded in three distinct phases: (1) PLR validation, (2) luminance washout and baseline, and (3) cognitive load assessment.

Phase 1: PLR Validation (60 Seconds)

This phase establishes sensor functionality and individual PLR dynamics through controlled luminance alternations. The entire visual field transitions between dark RGB (20,20,20) and bright RGB (180,180,180) conditions across three cycles, with each state maintained for 10 seconds. No auditory stimuli are presented to isolate purely visual responses.

Phase 2: Washout and Baseline (60 Seconds)

A 60-second washout period enables luminance adaptation and eliminates residual PLR effects. This duration was selected to ensure complete dissipation of residual constriction before the cognitive phase. The visual environment transitions to the iso-luminant target: background RGB (90,90,90) with a central fixation cross at RGB (110,110,110). The final 10 seconds serve as the luminance-adapted baseline.

Phase 3: Cognitive Load Blocks (240 Seconds)

The core manipulation occurs during this phase, wherein cognitive load is modulated exclusively through auditory tasks while the visual environment remains absolutely constant. This ensures that any observed pupillary changes reflect cognitive processing rather than luminance-driven reflexes.

The phase comprises four 45-second task blocks arranged in an ABBA design (Low-High-High-Low) to mitigate temporal order effects. The sequence proceeded as Low Load (45 s) → High Load (45 s) → High Load (45 s) → Low Load (45 s), separated by 15-second rest intervals.

Low-load blocks: Involve passive listening to single-digit numbers (conducted in Turkish) presented at 2-second intervals (participants mentally repeat each digit).

High-load blocks: Employ 1-back mental arithmetic wherein participants continuously sum each presented digit with the previous digit ((n-1) + n).

All auditory stimuli are pre-generated Text-to-Speech (TTS) recordings with consistent timing, volume, and tonal characteristics to eliminate acoustic confounds.

Signal processing

Raw pupil data undergoes dual-pathway preprocessing. Initial data cleaning involved artifact detection and removal. Artifacts were identified as rapid diameter changes exceeding ±10 mm/s or intervals of zero-value data. These segments were dilated by 50 ms to capture marginal artifacts and replaced using linear interpolation. A data loss threshold of 15% was established as an exclusion criterion to ensure reliable parameter estimation while accommodating natural blink rates; however, no sessions exceeded this limit, and thus all 22 sessions were included in the final analysis. For PLR analysis, minimal filtering (4 Hz low-pass Butterworth) preserves rapid reflexive dynamics. For cognitive analysis, aggressive filtering (0.5 Hz low-pass) isolates sustained cognitive trends from saccadic noise [11]. The use of differential filtering introduces a methodological asymmetry but is justified by the distinct temporal frequencies of the two physiological responses; applying the 0.5 Hz filter to the PLR would artificially flatten its peak velocity, while applying 4 Hz to the cognitive signal would introduce noise that mimics dilation velocity.

Outcome metrics

PLR Metrics

Amplitude (mm) = mean dark diameter − mean bright diameter; constriction percentage; peak constriction velocity (mm/s) = maximum negative first derivative during bright-phase onset.

Cognitive Load Metrics

Difference (mm) = mean high-load diameter − mean low-load diameter; percent change; peak dilation velocity (mm/s) = maximum positive first derivative during high-load onset.

Temporal Validation

Velocity ratio = PLR constriction velocity / cognitive dilation velocity. Values substantially >1.0 confirm temporal dissociation between reflexive and cognitive responses, validating that the iso-luminant paradigm successfully isolates TEPR from PLR.

Results

A total of 22 experimental sessions were analyzed. Summary statistics are presented in Table 1.

**Table 1:** Summary Statistics and Statistical Validation (N = 22 Sessions) P-values from one-sample t-test (PLR amplitude ≠ 0, cognitive diff. ≠ 0) and Wilcoxon signed-rank test (velocity ratio > 1). Effect size: Cohen's d. PLR: pupillary light reflexes; SD: standard deviation

Metric	Mean (SD)	Range	Test Statistic	P-value	Effect Size (d)
PLR Amplitude (mm)	1.52 (0.33)	1.07–2.22	t(21)=21.60	≤.001	4.60
Cognitive Diff. (mm)	0.38 (0.28)	0.07–1.18	t(21)=6.36	≤.001	1.37
Velocity Ratio	5.17 (2.85)	1.18–13.9	Z=4.11	≤.0001	-

While Table 1 summarizes the key validation metrics with statistical significance, Table 2 presents the complete parameter distributions for both PLR and cognitive load measurements. 

**Table 2:** Detailed PLR and Cognitive Load Metrics (N = 22 Sessions) PLR: pupillary light reflexes; SD: standard deviation

Metric	Mean	SD	Range
PLR Metrics (Phase 1)			
Dark baseline diameter (mm)	4.75	0.82	3.75–6.80
Bright constricted diameter (mm)	3.23	0.57	2.41–4.87
Constriction percentage (%)	31.7	3.9	25.4–41.1
Peak constriction velocity (mm/s)	19.1	11.2	4.9–47.1
Cognitive Load Metrics (Phase 3)			
Low-load mean diameter (mm)	4.59	1.03	2.92–7.27
High-load mean diameter (mm)	4.97	1.20	2.99–8.04
Cognitive change (%)	8.0	5.3	2.3–20.5
Peak dilation velocity (mm/s)	3.86	1.44	1.64–7.15

PLR Validation

All 22 sessions demonstrated robust PLR responses, confirming adequate sensor sensitivity. Mean amplitude was 1.52 ± 0.33 mm (31.7% constriction). Peak constriction velocity averaged 19.1 ± 11.2 mm/s, consistent with established brainstem-mediated PLR dynamics [4,12].

Cognitive Load Discrimination

Under iso-luminant conditions, significant pupillary differences emerged between load conditions. Mean diameter during low-load passive listening was 4.59 ± 1.03 mm, increasing to 4.97 ± 1.20 mm during high-load arithmetic - a 0.38 ± 0.28 mm difference (8.0% increase), confirming consistent TEPR detection (t(21)=6.36, p < .001).

Temporal Dissociation: The Critical Validation

The velocity ratio averaged 5.17 ± 2.85 across sessions, with all sessions exceeding 1.0. This five-fold difference in response velocity is the key finding: PLR-mediated constriction occurs rapidly (19.1 mm/s mean) through the pretectal-Edinger-Westphal pathway, while cognitive load-mediated dilation occurs gradually (3.86 mm/s mean) through the LC-NE pathway [2,5]. This temporal signature difference, consistent with the established neuroanatomy, provides direct evidence that the pupillary changes observed under iso-luminant conditions reflect cognitive processing rather than residual luminance effects. The gradual nature of cognitive dilation is not merely smaller in magnitude but fundamentally different in temporal dynamics, confirming successful isolation of TEPR.

## Discussion

This technical report validates an iso-luminant framework that successfully disentangles cognitive load responses from luminance-driven reflexes. The key innovation lies in eliminating the confound at the source rather than attempting post-hoc correction.

Traditional approaches employ luminance monitoring and regression-based correction [9,10]. These methods assume linear PLR responses and stable dynamics, assumptions often violated in complex VR environments. Furthermore, individual PLR variability introduces substantial inter-subject variance that population-level corrections inadequately address [13]. The iso-luminant approach circumvents these limitations entirely by ensuring zero luminance-driven modulation during cognitive assessment.

The velocity ratio metric provides novel quantitative validation. PLR operates through the pretectal olivary nucleus and Edinger-Westphal nucleus, producing rapid dynamics (typically <1 second) [4]. Cognitive load modulates pupil diameter through the LC-NE system, producing gradual dilations over 1-3 seconds [5,6,14]. Our observed five-fold velocity difference aligns precisely with this neuroanatomical distinction. Critically, this temporal signature cannot be explained by residual luminance effects, as the visual environment remained absolutely constant during cognitive assessment. The gradual, sustained nature of cognitive dilation, as opposed to rapid, reflexive PLR, constitutes independent validation that the iso-luminant paradigm successfully captures authentic TEPR.

Beyond laboratory validation, this iso-luminant framework has significant clinical potential, though the current study is exploratory in this regard. The LC-NE system dysfunction is implicated in several neuropsychiatric and neurodegenerative conditions. In ADHD, dysregulated noradrenergic signaling affects sustained attention and cognitive control [15,16]. While we did not test clinical populations, pupillometric measures of cognitive load could theoretically provide objective biomarkers for ADHD assessment and treatment monitoring, complementing subjective symptom scales. Similarly, neurodegenerative diseases, including Alzheimer's and Parkinson's disease, exhibit early LC-NE system pathology [17,18]. The isolated TEPR signal enabled by this framework could serve as a sensitive, non-invasive marker for detecting subtle cognitive changes before clinical symptom onset.

Limitations

The iso-luminant constraint restricts the paradigm to tasks without visual information processing. The current implementation uses a single luminance level; future work might explore whether different iso-luminant values produce comparable TEPR. Additionally, the absence of behavioral performance metrics (e.g., response accuracy) limits the direct validation of participant engagement and cognitive load manipulation. Furthermore, as the LC-NE system mediates both cognitive load and general arousal, the observed pupillary dilations likely reflect a composite of these processes; while our framework successfully isolates central autonomic responses from peripheral light reflexes, disentangling specific cognitive load from general arousal remains a challenge for future designs. Finally, the study implies a need for larger sample sizes and calculation of demographic effects; the lack of detailed demographic control variables (age, gender, background) is a limitation. The Turkish-language stimuli may also limit direct cross-cultural generalization.

## Conclusions

This study validates an iso-luminant VR framework that successfully isolates TEPR from PLR. Two key findings support this conclusion: (1) Cognitive load produced significantly smaller pupillary changes compared to the robust response observed during the PLR validation phase, and (2) the temporal dynamics exhibited a marked dissociation; cognitive dilation occurred gradually, whereas PLR constriction was rapid.

This resulted in a substantial velocity ratio that is consistent with distinct neural pathways. This temporal dissociation, the gradual nature of cognitive-load-induced dilation versus rapid light-induced constriction, provides direct evidence that under iso-luminant conditions, pupil diameter changes reflect authentic cognitive effort through the LC-NE pathway. The methodology establishes a reproducible foundation for VR-based neuro-ergonomic research. We frame this framework primarily as a subject-level validation tool, with potential applicability in clinical research, such as ADHD assessment and early detection of neurodegenerative disease.
